# Children with Iron Deficiency Anemia Have a Tendency to Hypercoagulation: An Evaluation by Thromboelastography

**DOI:** 10.4274/tjh.galenos.2019.2019.0027

**Published:** 2020-02-20

**Authors:** Ceren Kılcı, Lale Olcay, Beril Özdemir, Ali Fettah, Meriç Yavuz Çolak

**Affiliations:** 1 Başkent University Faculty of Medicine, Department of Pediatrics, Ankara, Turkey; 2 Başkent University Faculty of Medicine, Department of Pediatrics, Department of Pediatric Hematology-Oncology, Ankara, Turkey; 3 Sami Ulus Pediatrics Training and Research Hospital, Department of Pediatric Hematology-Oncology Ankara, Turkey; 4 Başkent University, Faculty of Health Sciences, Ankara, Turkey

**Keywords:** Thromboelastograph, TEG, Iron deficiency anemia, coagulation, Thromboembolism, Fibrinogen, Platelet functions

## To the Editor,

In the literature, there are many reports about patients who developed thrombosis with coexistent iron deficiency (ID) or ID anemia (IDA) [1,2,3,4,5,6,7,8].

Moreover, the frequency of severe anemia [1] and IDA [2] in patients who developed cerebral venous thrombosis (CVT) or deep venous thrombosis (including pulmonary embolism), respectively, was shown to be higher than in controls. The occluded vessels were cerebral vessels in 96.2% and 46.4% of the affected children and adults, respectively [8].

The predilection to hypercoagulation in ID/IDA was predicted to be due to reactive thrombocytosis, microcytosis, dehydration, infections, alterations in laminar flow, formation of turbulence, corruption of the oxidant/antioxidant balance, increases in platelet aggregation, increased procoagulants, and hypoxia [3,4,5,6,7,8]. However, laboratory investigations of this topic are still rare.

Herein we aim to provide laboratory evidence of the propensity to thrombosis in IDA using thromboelastography, which can qualitatively determine the status of coagulation as hyper- or hypocoagulation, and to state whether the abnormality stems from any pathology in primary hemostasis, secondary hemostasis, or the fibrinolytic system or any effects of anticoagulants or inhibitors within 30 min. With thromboelastography, the formation, strength, elasticity, and firmness of a clot can be shown using parameters such as reaction (R) time, clot formation (K) time, alpha (α) angle, maximum amplitude (MA), maximum lysis (LY30), and coagulation index (CI). Their functions and implications are presented in Supplemental Table 1.

Blood samples from 34 IDA patients between the ages of 3.5 and 191 months and from 39 healthy children of 12 to 191 months of age were studied using the flat cup test in thromboelastography (TEG ^®^ 5000 Thromboelastograph® Hemostasis Analyzer).

Patients with chronic (including thalassemia) or infectious/inflammatory diseases, high c-reactive protein (CRP) levels, obesity, hypertension, smoking habit, hyperuricemia, liver or renal function abnormalities, vitamin B12 or folic acid deficiencies, and self or family history of thrombosis or bleeding were excluded from the study, as were those on any drug therapy.

The thromboelastographic measurements observed in the IDA and control groups were as follows: K, 1.4±0.6 vs. 1.8±1.1 min (p=0.03); MA, 70.6±4.9 mm vs. 66.9±8.3 mm (p=0.05); LY30, 3.8±4.4 vs. 2.0±3.2 (p=0.12); R, 3.9±1.4 vs. 4.0±1.4 min (p=0.78); α, 53.0±8.9° vs. 53.0±9.6° (p=0.91); and CI, 1.0±1.4 vs. 0.3±2.1 (p=0.19) (Supplemental Table 2; Figures 1A and 1B).

Significant decrease in K and increase in MA with borderline significance compared to the controls implied hypercoagulability, which was possibly due to increased fibrinogen levels and/or to a lesser extent increased thrombocyte functions (Supplemental Table 1). Inflammation-related hyperfibrinogenemia was a remote possibility since patients with infection/inflammation and high CRP levels were excluded; however, we could not establish fibrinogen levels and thrombocyte functions. Other studies showed normal levels of fibrinogen [9] and increased [10] or decreased thrombocyte aggregation [11] in IDA.

Our findings revealed a positive linear relationship between serum iron levels and α (p=0.034; r=0.339) and between red blood cell distribution width (RDW) and α (p=0.004; r=0.448), and an inverse linear relationship between RDW and K (p=0.048; r=-0.319) in the control group.

In the IDA group, there was a positive and weak linear relationship between ferritin and α (p=0.049; r=0.341), a positive linear relationship between mean corpuscular volume (MCV) and MA (p=0.04; r=0.353), and an inverse linear relationship between thrombocyte count and K (p=0.041; r=-0.353).

Although the positive linear relationship of ferritin with α and of MCV with MA pointed at hypocoagulability, the inverse linear relationship between thrombocyte count and K pointed at hypercoagulation, being correlated with the severity of thrombocytosis, the latter of which is a usual finding in IDA. These conflicting results of the correlation studies may be due to the limited number of patients.

In another study similar to ours that investigated the effect of IDA by rotational thromboelastometry (ROTEM), normal coagulation test results were also revealed, whereby maximum clot firmness in ROTEM, equivalent to MA in thromboelastography, was increased in the IDA group and  clot formation time, equivalent to K in thromboelastography, was decreased in the IDA group, both implying hypercoagulability. This study also revealed similar thrombocyte counts in the IDA and control groups despite a negative correlation between thrombocyte count and CFT [equivalent to K in thromboelastography] [9], as in our study.

The fact that none of our patients in this report had developed thrombosis suggests that additional determinants may be required for the development of thrombosis. Moreover, the real incidence of thrombosis in IDA may be too low to be established in a small cohort of patients such as ours. The duration and the severity of anemia may be other factors for the initiation of thrombosis. In a review of 54 patients who developed thrombosis on the basis of ID/IDA, the majority had “severe” IDA [8], while Stolz et al. [1] reported “not mild” but rather “severe” anemia to be more frequent in patients with CVT than normal subjects and “severe anemia” was an independent determinant of CVT. ID was an independent predictor of venous thromboembolism recurrence [12]. In our study, due to the limited number of participants, we could not compare cases in terms of “severe” and “mild” anemia.

Indeed, of the 54 patients with coexistent ID/IDA and thrombosis in the literature [8], 18% had thrombotic risk factors and 75.9% had associated diseases/disorders [8], and both of our two patients with both IDA and thrombosis had thrombotic risk factors (100%) and an associated condition (100%). Since not all patients in the literature were evaluated for thrombophilia factors, this rate may be increased. We could not evaluate these patients for accompanying thrombophilic factors.

We furthermore did not have an opportunity to compare thromboelastography values before and after iron therapy. However, our findings showed a propensity to hypercoagulation in patients with IDA and confirm the results of previous studies [1,2,8,9].

Although further laboratory evaluations are required with larger numbers of patients and the exclusion of accompanying thrombophilic factors, IDA seems to be a new candidate among thrombotic factors.

## Figures and Tables

**Supplemental Table 1:**
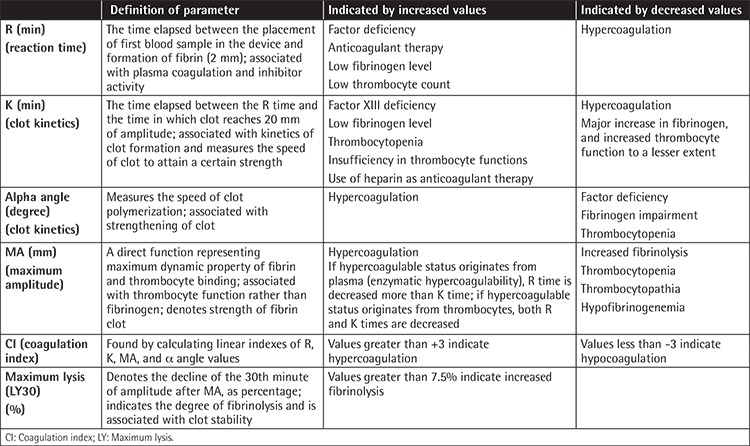
Definition of thromboelastographic parameters [1,2].

**Supplemental Table 2:**
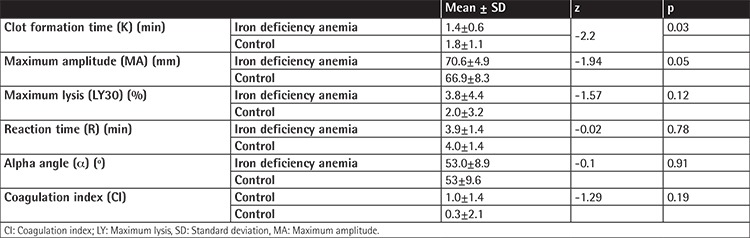
Thromboelastographic parameters in the iron deficiency anemia group in comparison with the healthy control group.

**Figure 1A:**
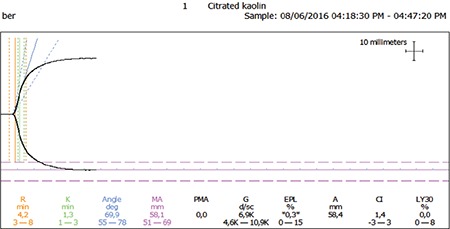
A normal thromboelastography result for an individual from the control group.

**Figure 1B:**
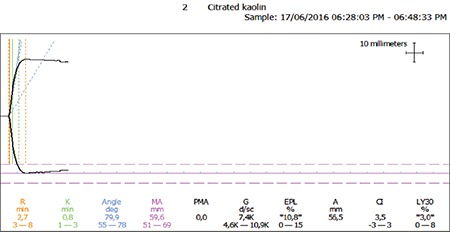
Thromboelastography result for a patient with iron deficiency anemia showing hypercoagulation.
